# Induction of oxidative- and endoplasmic-reticulum-stress dependent apoptosis in pancreatic cancer cell lines by DDOST knockdown

**DOI:** 10.1038/s41598-024-68510-8

**Published:** 2024-09-02

**Authors:** Richard Böhme, Andreas W. Schmidt, Nico Hesselbarth, Guido Posern, Andrea Sinz, Christian Ihling, Patrick Michl, Helmut Laumen, Jonas Rosendahl

**Affiliations:** 1 Department of Internal Medicine I, Martin Luther University Halle-Wittenberg, Halle (Saale), Germany; 2 Institute of Medical Genetics and Applied Genomics, University Hospital Tübingen, Tübingen, Germany; 3 Paediatric Nutritional Medicine, Else Kröner Fresenius Center for Nutritional Medicine, Technical University of Munich (TUM), Freising, Germany; 4 Institute for Physiological Chemistry, Medical Faculty, Martin Luther University Halle-Wittenberg, Halle (Saale), Germany; 5 Department of Pharmaceutical Chemistry and Bioanalytics, Institute of Pharmacy, Martin Luther University Halle-Wittenberg, Halle (Saale), Germany; 6 Department of Internal Medicine IV, Heidelberg University, University Hospital Heidelberg, Heidelberg, Germany

**Keywords:** Pancreatic cancer, Pancreatic cancer

## Abstract

The dolichyl-diphosphooligosaccharide-protein glycosyltransferase non-catalytic subunit (DDOST) is a key component of the oligosaccharyltransferase complex catalyzing *N*-linked glycosylation in the endoplasmic reticulum lumen. DDOST is associated with several cancers and congenital disorders of glycosylation. However, its role in pancreatic cancer remains elusive, despite its enriched pancreatic expression. Using quantitative mass spectrometry, we identify 30 differentially expressed proteins and phosphopeptides (DEPs) after DDOST knockdown in the pancreatic ductal adenocarcinoma (PDAC) cell line PA-TU-8988T. We evaluated DDOST / DEP protein–protein interaction networks using STRING database, correlation of mRNA levels in pancreatic cancer TCGA data, and biological processes annotated to DEPs in Gene Ontology database. The inferred DDOST regulated phenotypes were experimentally verified in two PDAC cell lines, PA-TU-8988T and BXPC-3. We found decreased proliferation and cell viability after DDOST knockdown, whereas ER-stress, ROS-formation and apoptosis were increased. In conclusion, our results support an oncogenic role of DDOST in PDAC by intercepting cell stress events and thereby reducing apoptosis. As such, DDOST might be a potential biomarker and therapeutic target for PDAC.

## Introduction

Pancreatic ductal adenocarcinoma (PDAC) is a devastating disease, characterized by late diagnosis, early metastasis, limited response to chemotherapy and poor prognosis^1^. Despite significant advances in understanding the pathobiology of the disease in recent decades, PDAC is predicted to be the third leading cause of cancer related mortality in Europe by 2025^2^, in part reflecting the increasing prevalence of the risk factors obesity, diabetes and alcohol consumption, but also the lack of successful therapies^3^. PDAC arises from the exocrine tissue, which is characterized by a high protein expression and secretion capacity.

Secretory proteins undergo *N*-linked glycosylation during their endoplasmic reticulum (ER) transit. In this process, a pre-assembled core oligosaccharide can be attached to the asparagine (Asn) residue of the Asn-Xaa-Ser/Thr motif (Sequon) in the nascent polypeptide chain, by the oligosaccharyltransferase (OST) complex as it enters the ER lumen^4^. *N*-linked oligosaccharides can promote protein folding by increasing the stability of the unfolded polypeptide chain, preventing aggregation, and allowing cell surface glycoproteins to localize on the cell surface^5^. OST complexes catalyzing *N*-linked glycosylation consist of 12 proteins, including the STT3 OST complex catalytic subunit A and B (STT3A, STT3B), defender against cell death 1 (DAD1), ribophorin 1 (RPN1), ribophorin 2 (RPN2) and dolichyl-diphosphooligosaccharide–protein glycosyltransferase non-catalytic subunit (DDOST)^6^. As an important post-translational modification, *N*-linked glycosylation plays a critical role in the folding, stability, subcellular localization, and biological function of glycoproteins. Aberrant *N*-linked glycosylation has been widely recognized as an important characteristic of various cancers, such as colorectal, breast or liver cancer and correlates with tumor development, progression, metastasis, and chemo resistance^7–10^. Knockdown (KD) of drosophila DAD1 (dDAD1) or human RPN1 induces ER stress-dependent apoptosis, whereas expression levels of several OST subunits including RPN1, RPN2, STT3A STT3B, and DDOST were upregulated in breast cancer^11,12^. Interestingly, in PDAC cell lines, the glycolytic inhibitor 2-deoxy-D-glucose (2DG) reduces protein *N*-glycosylation and induces ER related apoptosis^13^. Of note, inhibiting the OST complex and thereby, *N*-linked glycosylation of proteins, was found to induce ER stress-dependent apoptosis^11,12^. In tumor cells, protein processing in the ER is often impaired either intrinsically, exemplarily by oncogenic activation, or extrinsically, by hypoxic, acidic and nutrient deprived milieu^14^. Consequently, the accumulation of misfolded proteins in the ER lumen results in ER stress. This induces the unfolded protein response (UPR) to enhance clearance capacities and thus restore ER homeostasis. Although the UPR is an important cytoprotective response, prolonged ER stress can nevertheless lead to apoptosis^14,15^.

A recent study compared the expression of DDOST between gliomas and normal brain tissue in the Gene Expression Omnibus (GEO) and Chinese Glioma Genome Atlas (CGGA) databases. In glioma patients, high levels of DDOST correlated with aggressiveness and an altered immunosuppressive microenvironment^16^. In hepatocellular carcinoma (HCC), high DDOST expression was found to be associated with poorer overall and disease-specific survival of HCC patients^17^. Besides the well-established function as OST-complex subunit, DDOST was identified as a potential receptor for advanced glycation end products (AGE-R1)^18^ and as such, acted as suppressor for cell oxidant stress and activation signaling via the epidermal growth factor receptor (EGFR) in mesangial and embryonic kidney cells^19^.

In summary, DDOST expression has been shown to be relevant in distinct cancers, but little is known about the function of DDOST in the development and progression of PDAC from functional studies or public domain databases. Here, we demonstrate that DDOST affects several biological processes important for proliferation, oxidative stress and apoptosis at the proteome and phosphoproteome level in PDAC cell lines. We also show that oxidative and ER stress-induced cell apoptosis inhibits cell viability after DDOST KD.

## Results

### Proteomic analysis in PDAC cells identifies differentially expressed and phosphorylated proteins upon DDOST KD

**Figure 1:**
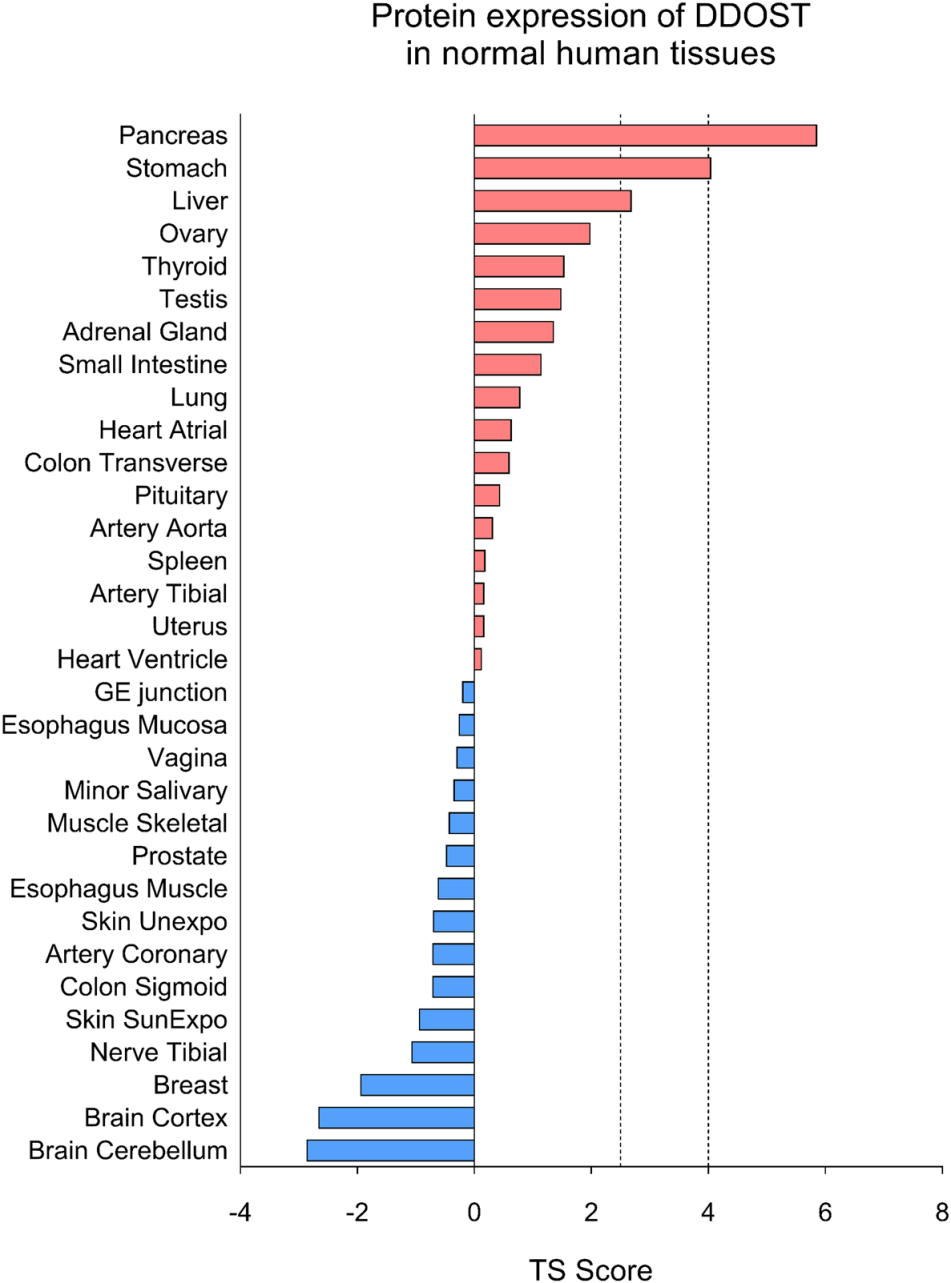
Tissue Specificity Scores of DDOST protein expression in human body donors. If a gene has TS (tissue specificity) scores at least in one tissue ≥ 2.5, this gene is called tissue-enriched. Vertical lines indicate the threshold values of 2.5 and 4. Adapted from “A Quantitative Proteome Map of the Human Body” by Jiang et al., 2020.

**Figure 2:**
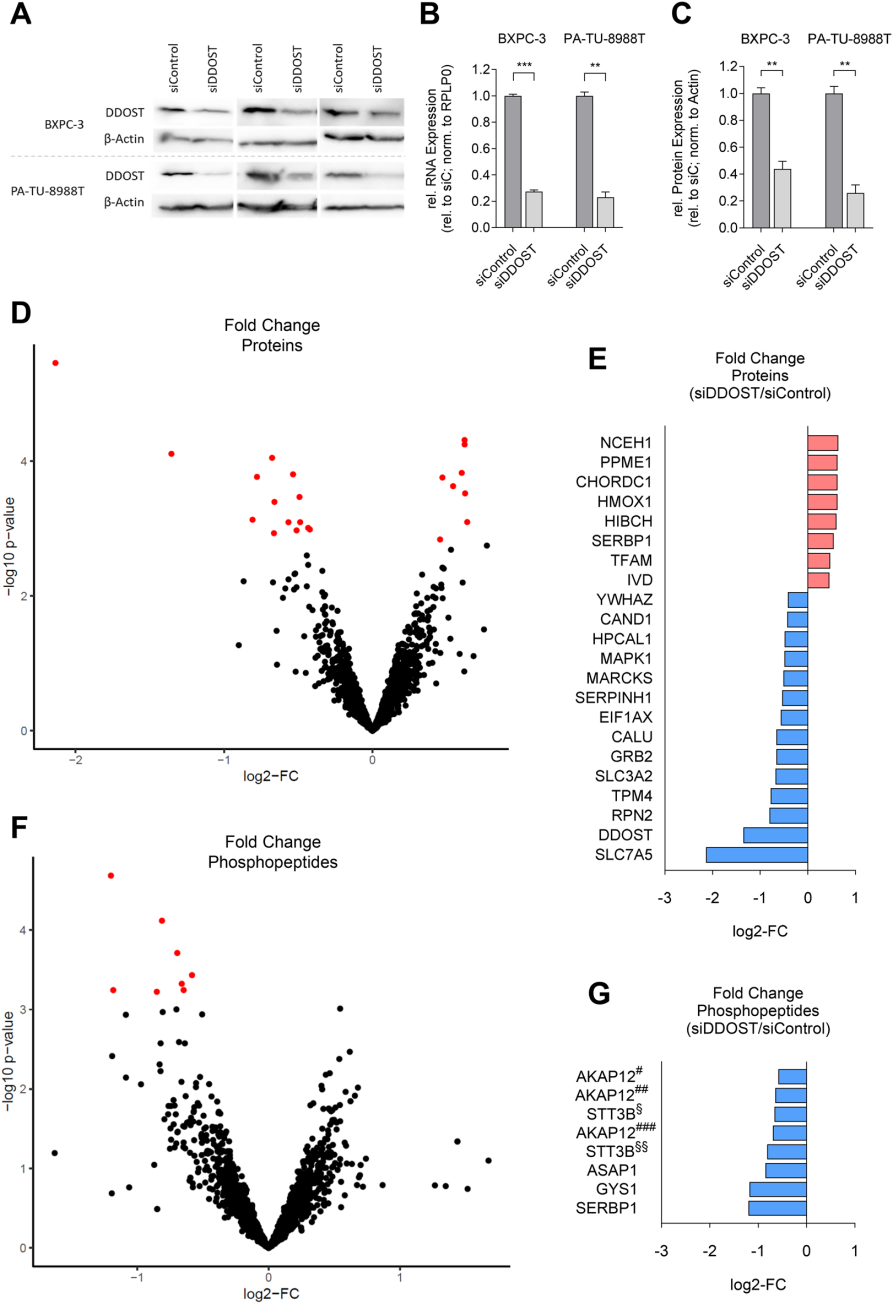
Fold change (FC) of total proteins and phosphopeptides after DDOST KD in PA-TU-8988T cell line. (**A**) Western blot analysis of DDOST expression after DDOST KD. β-Actin was used as loading control. (**B**, **C**) Quantification of rel. RNA expression and rel. protein expression levels of DDOST after KD (**P < 0.01, ***P < 0.001; unpaired *t*-test). (**D**, **F**) Volcano plot of protein and phosphopeptide log2-FC. Highlighted proteins and phosphopeptides cutoff FDR < 0.05 (n = 5; ROTS-test). (**E**, **G**) Bar chart of protein and phosphopeptide log2-FC. Upregulated proteins and phosphopeptides in red, downregulated in blue.

Interestingly, a quantitative proteome map in healthy human body donors revealed that DDOST is tissue-specifically enriched in pancreas (Fig. 1), indicating an important role in pancreatic function and possibly a role in pancreatic tumorigenesis^20^. Moreover, in different PDAC tumor cell lines, ranging from BXPC-3 and PA-TU-8988T (Fig. 2A) to PANC-1 (Fig. S1) we found DDOST protein expression in western blot analysis consistently.

To unravel potential regulative effects on the proteome, including the phosphoproteome, by DDOST we performed KD experiments of DDOST in the PDAC cell line PA-TU-8988T followed by quantitative LC–MS/MS. Cells were each transiently transfected with two homolog-specific siRNA pools to KD DDOST expression. KD efficiency was analyzed by both, qRT-PCR and western blot analysis. DDOST expression was reduced successfully by an average of 75% in the cell lines PA-TU-8988T and BXPC-3 compared to a non-targeting control siRNA (*P* < 0.05, Fig. 2A–C). To identify all proteins and phosphopeptides regulated by DDOST in the pancreatic cancer cell line PA-TU-8988T, we performed a quantitative proteome analysis by TMT-labeling and LC–MS/MS, including an additional phosphopeptide enrichment step, after DDOST KD. In summary, 1577 proteins and 2059 phosphopeptides in 883 proteins were identified (Supplemental Table S1; Supplemental Table S2). The expression levels of eight proteins were significantly increased, while 14 proteins and eight phosphopeptides in five proteins were significantly decreased (FDR < 0.05, Fig. 2D–G). Notably, besides DDOST, two additional OST complex DEPs were down-regulated, the protein RPN2 (Ribophorin II) (FDR = 0, log2-FC = − 0.81) and two phosphopeptides of the protein STT3B (STT3 Oligosaccharyltransferase Complex Catalytic Subunit B) (FDR = 0, log2-FC = − 0.81 / − 0.66).

### Protein–protein interaction analysis provides information on correlations between identified proteins and phosphopeptides in public domain databases

**Figure 3:**
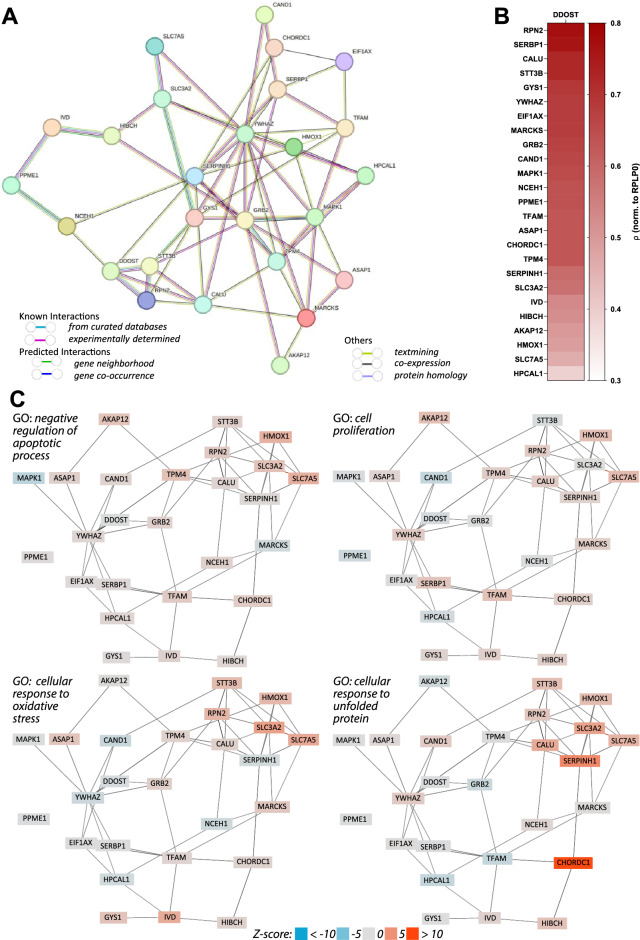
Interactions and correlations of 26 proteins identified as DEPs. (**A**) PPI network consisting of 26 nodes and 67 edges from STRING database (PPI enrichment *P* = 2.83 × 10^−4^). (**B**) Spearman correlation analysis of mRNA levels (TCGA pancreatic adenocarcinoma) comparing *DDOST* with all identified DEPs (*P* < 1.00 × 10^−4^; n = 179). (**C**) Functional enrichment analysis as implemented in the GADO^23^ webserver (negative regulation of apoptotic process: *P* = 2.54 × 10^−6^; cell proliferation: *P* = 5.35 × 10^−4^; cellular response to oxidative stress: *P* = 1.11 × 10^−3^; cellular response to unfolded protein: *P* = 1.18 × 10^−3^).

A functional network of differentially expressed proteins and phosphopeptides (DEPs, Supplemental Table S3) was constructed from the STRING database to evaluate known and potential protein–protein-interactions (PPI). The resulting PPI network consisted of 26 nodes and 67 edges, with each node representing all splice isoforms or post-translational modifications of each analyzed DEP and each edge representing all predicted or functional associations. The resulting network contains significantly more interactions than expected for a random group of proteins of the same size and degree distribution from the genome (PPI enrichment *P* = 2.83 × 10^−4^, Fig. 3A).

Next, we performed a spearman correlation analysis of *DDOST* mRNA-expression levels with each of the identified DEPs in 179 pancreatic adenocarcinoma (PAAD) tumor tissue samples (Fig. 3B). We found the strongest correlations between *DDOST* and *RPN2* (*ρ* = 0.77, *P* < 1.00 × 10^−4^), *SERBP1* (*ρ* = 0.76, *P* < 1.00 × 10^−4^), *CALU* (*ρ* = 0.72, *P* < 1.00 × 10^−4^), *STT3B* (*ρ* = 0.72, *P* < 1.00 × 10^−4^), *YWHAZ* (*ρ* = 0.68, *P* < 1.00 × 10^−4^), *MAPK1* (*ρ* = 0.65, *P* < 1.00 × 10^−4^). Additionally, 16 significant correlations with *ρ* ≥ 0.5 (*P* < 1.00 × 10^−4^) and 3 correlations with *ρ* ≥ 0.39 (*P* < 1.00 × 10^−4^) were detected (Supplemental Table S3).

### GO annotation analyses identifies biological processes enriched for DEPs.

To determine biological processes in which the here identified DEPs may participate, we assessed functional enrichments in the network of DEPs using STRING (Cellular Components, Gene Ontology)^21,22^. We found two complexes enriched, including five of our identified DEPs, the OST complex (FDR = 2.80 × 10^−3^ included DEPs: RPN2, STT3B, DDOST) and the amino acid transport complex (FDR = 0.01; included DEPs: SLC7A5, SLC3A2) (Supplemental Table S4), suggesting an impact of DDOST on the OST complex function.

Next, we applied the function enrichment analysis of the GADO tool^23^ using gene co-regulation to improve prediction of pathway membership, to the here identified DEPs. We found a significant enrichment of the protein glycosylation pathway (*P* = 0.02, Table S5, highlighted in red), further supporting a potential impact on protein glycosylation by inference with the OST complex. Moreover, we also found numerous biological processes related to carcinogenesis significantly enriched (Supplemental Table S5, highlighted in red), ranging from *negative regulation of apoptotic process*, *cell proliferation, cellular response to oxidative stress* and *response to unfolded protein* (*P* = 2.54 × 10^−6^, *P* = 5.35 × 10^−4^, *P* = 1.12 × 10^−3^, *P* = 1.18 × 10^−3^, respectively, Fig. 3C and Supplemental Table S5) to *response to endoplasmic reticulum stress* (*P* = 0.03, Supplemental Table S5), suggesting an impact of deregulated OST complex by DDOST KD on typical phenotypes, that are common for tumor development.

### Phenotypical assays verify effects of DDOST KD on proliferation, viability, ER-stress, oxidative stress and apoptosis in PDAC cell lines

We performed DDOST KD experiments in the PDAC cell lines BXPC-3 and PA-TU-8988T to assess the impact on cell growth, viability, ER stress, ROS-formation and apoptosis. Tunicamycin (TM) as inhibitor of *N*-linked glycosylation was used as positive control in some experiments, to estimate potential effect maxima.

#### DDOST KD reduces proliferation and viability in PDAC cells

**Figure 4:**
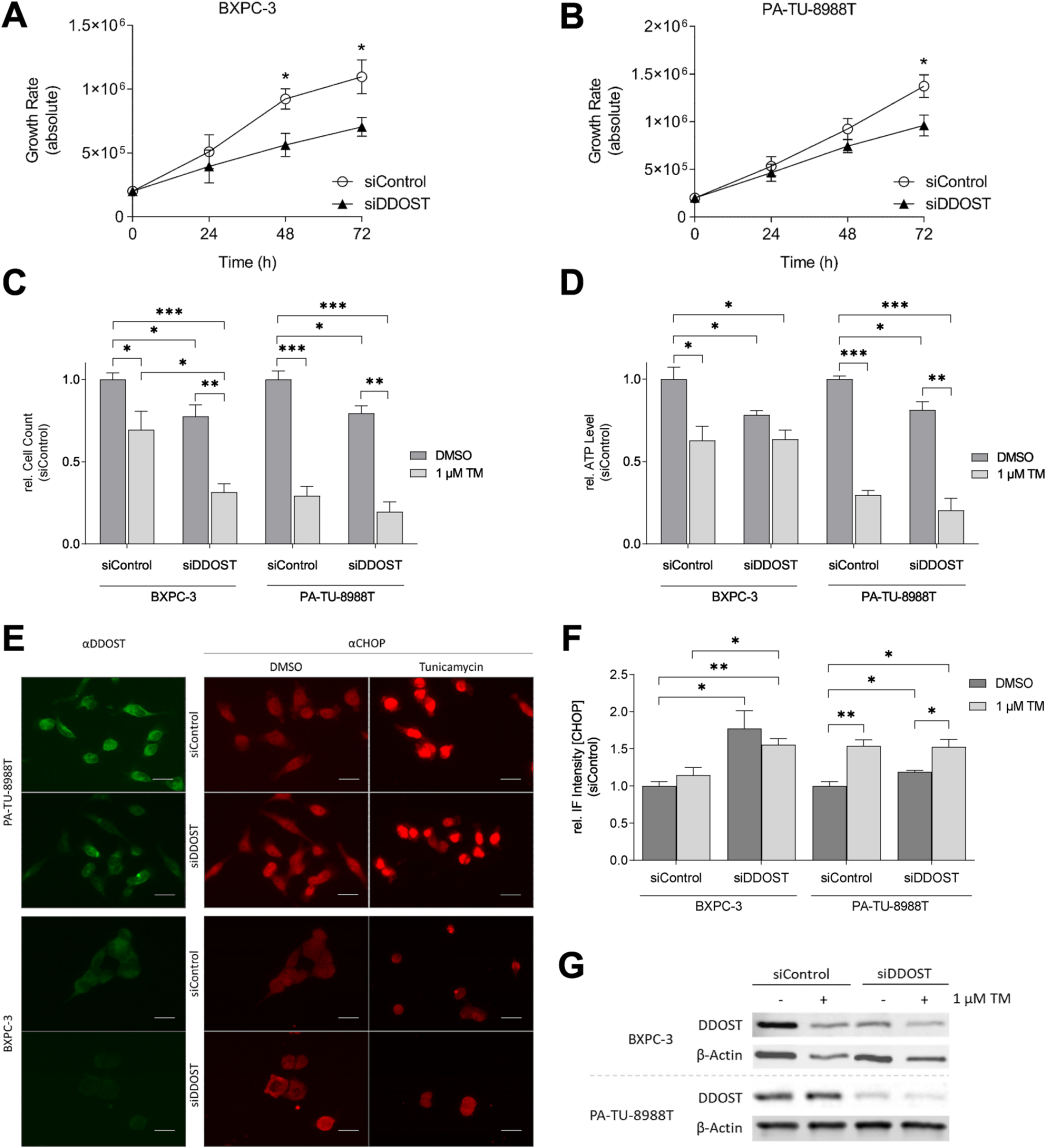
Reduced proliferation and viability and induced ER stress after DDOST KD in PDAC cell lines. (**A**) Growth curve of BXPC-3 cells after DDOST KD. (**B**) Growth curve of PA-TU-8988T cells after DDOST KD. (**C**) Quantification of proliferation assay 72 h after DDOST KD and TM treatment. (**D**) Quantification of viability assay 72 h after DDOST KD and TM treatment. (**E**) Immunofluorescence images of DDOST and CHOP after DDOST KD and treatment with TM (scale bar = 50 µm). (**F**) Quantification of CHOP rel. IF intensity after DDOST KD and treatment with TM. (**G**) Western blot analysis of DDOST KD efficiency. β-Actin was used as loading control. (*P < 0.05, **P < 0.01, ***P < 0.001; unpaired *t*-test).

A cell growth assay over 72 h showed a DDOST KD dependent reduction in proliferation in both cell lines, BXPC-3 and PA-TU-8988T, ranging from 20 to 22% (*P* = 0.03 and *P* = 0.04, respectively, Fig. 4A–C). Furthermore, detection of the cellular ATP-level indicated a reduced viability of 19–22% in both cell lines after DDOST KD (*P* = 0.05 and *P* = 0.02, Fig. 4D). Treatment of siControl cells with 1 µM TM as a positive control for OST complex inhibition, led to a decrease in proliferation, ranging from 31 to 71% (*P* = 0.03 and *P* = 3.00 × 10^−4^, respectively, Fig. 4C) and decreased ATP-levels of 37–70% (*P* = 0.03 and *P* = 1.00 × 10^−4^, respectively, Fig. 4D). Moreover, treatment of siDDOST cells with 1 µM TM for 24 h after KD resulted in a 46–60% decrease in cell growth (*P* = 6.10 × 10^−3^ and *P* = 1.30 × 10^−3^, respectively, Fig. 4C) in both, BXPC-3 and PA-TU-8988T. Additionally, treatment of siDDOST cells with 1 µM TM led to 61% decrease in ATP level in PA-TU-8988T (*P* = 0.02, respectively, Fig. 4D) but not in BXPC-3. Thus, KD of DDOST reduces proliferation and cell viability in both tested cells lines, which is also observed for the OST complex inhibitor TM.

#### DDOST KD induces ER stress in PDAC cells

To examine the effects of DDOST KD on ER-related cell stress, protein level of the ER stress regulator CHOP was detected using an immunofluorescence assay. In BXPC-3, CHOP level was 77% increased and in PA-TU-8988T 19% increased after 48 h of DDOST KD (*P* = 0.03, *P* = 0.04, respectively, Fig. 4E,F). Treatment of siControl cells with 1 µM TM led to a 54% increase of CHOP level in PA-TU-8988T (*P* = 5.70 × 10^−3^, respectively, Fig. 4E,F) but not in BXPC-3. Additionally, treatment of siDDOST cells with 1 µM TM led to 34% increased CHOP level in PA-TU-8988T (*P* = 0.03, respectively, Fig. 4E,F) but not in BXPC-3. The ER stress level was increased in both tested cell lines after DDOST KD or TM treatment. Efficiency of DDOST KD was validated by western blot analysis (Fig. 4G).

#### DDOST KD induces oxidative stress in PDAC cells

**Figure 5:**
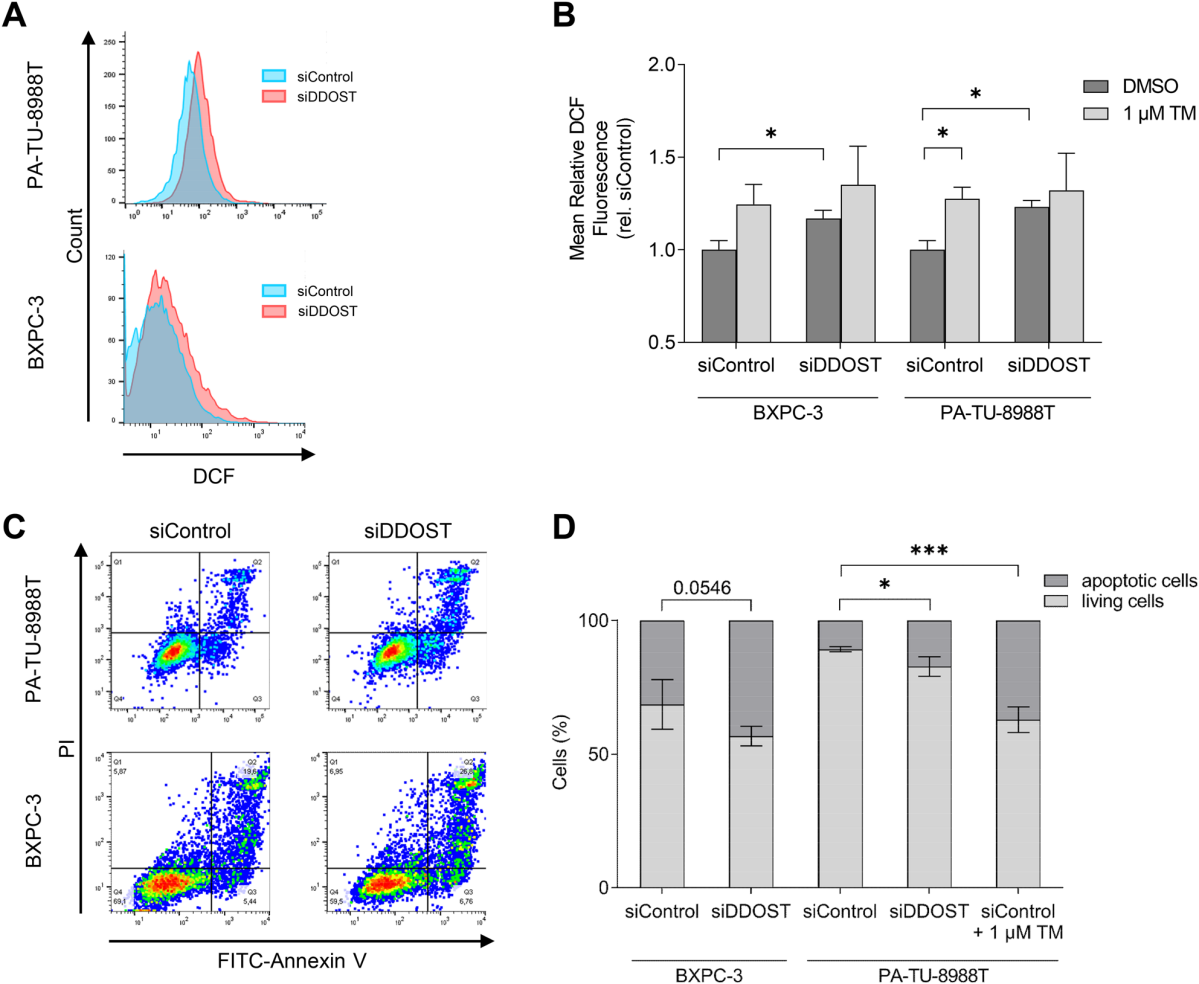
Induced ROS formation and Apoptosis after DDOST KD in PDAC cells. (**A**) Flow cytometry assay of ROS formation by DCF detection after DDOST KD. (**B**) Quantification of mean rel. DCF intensity after DDOST KD and treatment with TM. (**C**) Flow cytometry assay of FITC-Annexin V and PI staining after DDOST KD. (**D**) Quantification of FITC-Annexin V and PI positive cells after DDOST KD and treatment with TM. (*P < 0.05, ***P < 0.001; unpaired *t*-test). Western blot analysis of DDOST KD efficiency shown in Fig. 4G.

ROS formation assay was performed to assess the potential effects of DDOST KD on oxidative stress levels in PDAC cells. Therefore, cells were incubated with H_2_DCF-DA acting as indicator of intracellular ROS by its oxidized and fluorescent form DCF. Fluorescence intensity was determined 48 h post KD transfection using flow cytometry analysis. DCF intensity was increased 17% in BXPC-3 and 23% in PA-TU-8988T cell lines after DDOST KD (*P* = 0.04, *P* = 0.01, respectively, Fig. 5A,B). Further, treatment of siControl cells with 1 µM TM led to 28% increased DCF intensity in PA-TU-8988T (*P* = 0.02, respectively, Fig. 5B), but not in BXPC-3 cell line. Thus, the oxidative stress level was increased after KD DDOST and partially after TM treatment.

#### DDOST KD induces apoptosis in PDAC cells

To examine apoptotic effects of DDOST KD, PDAC cell lines were stained with Propidium Iodide (PI) and Annexin V to determine apoptotic activation using flow cytometry analysis 48 h post transfection. PI and Annexin V level were significantly increased by 62% in PA-TU-8988T but not significantly increased by 28% in BXPC-3 cells after DDOST KD (*P* = 0.01, *P* = 0.05, respectively, Fig. 5C,D). Further, treatment of siControl cells with 1 µM TM led to threefold increased PI and Annexin V level in PA-TU-8988T cells (*P* = 2.00 × 10^−4^, respectively, Fig. 5D). Thus, apoptosis was increased after DDOST KD or TM treatment in PA-TU-8988T but not in BXPC-3 cell line.

## Discussion

The pancreatic gland has a high secretory capacity and therefore requires a high level of protein translation, post-translational modification and secretion. The OST complex is essential for *N*-linked glycosylation, one of the most common protein modifications, and has been implicated in ER stress-induced cell death in PDAC^13,24^. Moreover, it has been shown that *N*-linked glycosylation of critical proteins is essential for tumorigenesis, proliferation and metastasis by maintaining cell homeostasis^9,12,25–27^.

The assembly and stability of the OST complex requires the subunit DDOST^28^, which has been correlated with immune infiltration, metastasis and prognosis in several types of cancer^16,17,29^. Additionally, data from a proteomic study suggested that DDOST function is particularly relevant in the pancreas, due to its tissue-specific expression^20^. However, no association with pancreatic cancer has been reported so far. As there are limited therapeutic tools for PDAC, new insights into relevant processes of PDAC development may contribute to the understanding of tumorigenesis and thereby help to open novel diagnostic and therapeutic avenues. The present study aims to determine how DDOST may affect PDAC cellular phenotypes. We performed KD of DDOST in human PDAC cell lines and used a mass-spectrometry based assay to detect DEPs. After matching these results to biological processes, they were phenotypically verified. We could experimentally validate our findings in phenotypic assays, finding a significant regulation of proliferation, oxidative stress, and apoptosis by DDOST KD.

Approximately half of human proteins are glycoproteins, the majority of which are *N*-linked glycosylated^30^. Protein *N*-linked glycosylation has been linked to skin cancer^31^, along with its crucial role in many cellular activities, including protein folding, stability and interaction^32^. A recent study reported that the expression level of the *amino acid transport complex* was significantly coregulated in head and neck squamous cell carcinoma tissues and cell lines^33^. An upregulation was associated with poor survival of oral squamous cell carcinoma patients. After KD of SLC3A2 reduced migration, invasion and proliferation but increased apoptosis in cancer cell lines was observed^34^. These results are consistent with our finding of reduced SLC3A2 expression together with reduced proliferation and increased apoptosis after KD of DDOST. In addition, we found that SLC7A5 expression was reduced after DDOST KD, and both SLC3A2 and SLC7A5 have been annotated to the *amino acid transport complex* by GSEA.

To analyze the relationships of the identified DEPs, we used the STRING database obtaining a PPI network including all significant regulated hits with a wide variety of interactions, of which one was the OST complex cluster with three identified proteins DDOST, RPN2 and STT3B. Interestingly, CALU, directly interacting with DDOST and RPN2, is known to be highly expressed in tumor cells and therefore might play a crucial role in cancer progression and the induction of epithelial-to-mesenchymal transition^35^. Furthermore, the protein with the most edges to other DEPs was YWHAZ, a central hub protein for many signal pathways frequently up-regulated in multiple types of cancers which is associated with cell growth, apoptosis, migration and invasion^36^. Another identified key regulator was MAPK1, which is known to regulate cell functions including proliferation, gene expression, differentiation, mitosis, cell survival, and apoptosis^37^. All 30 DEPs were significantly correlated with DDOST in 179 PAAD tumor tissue samples, among which 22 were strong correlations, including RPN2, SERBP1, CALU, STT3B, YWHAZ and MAPK1. The RNA-binding protein SERBP1 has been shown to play an important role in apoptosis and metabolic processes through post-transcriptional regulation of gene transcription and alternative splicing in HeLa cells, and to worsen the prognosis for PDAC survival^38^.

We found a significant association of the identified DEPs with carcinogenesis related pathways such as *negative regulation of apoptotic process* and *cell proliferation*, *cellular response to oxidative stress*, *response to endoplasmic reticulum stress* and *response to unfolded protein.* Previous studies have reported that dysregulation of the oxidative stress response play important roles in carcinogenesis and tumor progression by exploiting the respective response mechanisms under stress conditions^39,40^. To phenotypically validate our findings, we focused on the key biological processes annotated by GO analysis of the identified DEPs that are important for tumor development and further investigated them experimentally in vitro. In our study, we show that KD of DDOST by siRNA led to reduced proliferation rates and viability, as well as increased ER stress, ROS formation and apoptosis in PDAC cells. This is consistent with the finding that a mutation of DDOST causes a general defect in *N*-linked glycosylation leading to ER stress in gastric cancer cells^27,41^. A recent review, based on the effects of several therapeutics with the potential to increase ER stress, hypothesized that increased ER stress in pancreatic cancer activates the UPR and leads to ER-induced apoptosis via CHOP^42^. Additionally, increased levels of CHOP promoted ROS-induced apoptosis by mediating between ER stress signaling and ROS formation^43,44^. These reports are consistent with our finding that both ROS formation and apoptosis levels were increased after DDOST downregulation in PDAC cells.

Based on these data, we propose that DDOST has a tumor-promoting capacity in PDAC cells by maintaining ER homeostasis and thereby suppressing ROS formation and apoptosis. The proteomics inferred pathways could be functionally validated in the *KRAS*-mutant cell line PA-TU-8988T, yet in the *KRAS*-wild type cell line BXPC-3 we found similar effect however no significant impact on apoptosis. We have to note, that future, deeper studies are required to follow this initial observation. We detected 28 proteins, which rely on the expression of DDOST. However, the specific regulation of DDOST on non-glycosylated proteins requires further research. For this reason, the need for a glycosylation assay is a limitation since we cannot explain how DDOST affects non-glycosylated proteins or protein phosphorylation. An accumulation of un-glycosylated proteins may induce the UPR, explaining the observed effects. On the other hand, while MS analysis has allowed us to relate several proteins to DDOST, we suspect that several regulated genes were not identified by the mass spectrometric approach. Moreover, further studies are needed to clarify the exact relationship between DDOST and the candidate interaction partners identified in this study.

In conclusion, our in vitro experiments demonstrated that downregulation of DDOST induces ER stress leading to enhanced ROS formation and apoptosis, as well as reduced proliferation and cell viability in two human pancreatic cancer cell lines. Further, this study identified DEPs, which are related to DDOST and may be involved in pancreatic carcinogenesis. A total of 30 regulated proteins and phosphopeptides were identified and may be regarded as diagnostic biomarkers for PDAC. However, further studies are needed to elucidate the biological function of these proteins in PDAC.

## Methods

### Cell culture

PA-TU-8988T (RRID: CVCL-1847) and PANC-1 cells (RRID: CVCL-0480) were maintained in 90% Dulbecco’s MEM (Thermo Fisher Scientific), supplemented with 10% FBS, at 37 °C in humidified 5% CO_2_. BXPC-3 cells (RRID: CVCL_0186) were maintained in 90% RPMI 1640 (Thermo Fisher Scientific), supplemented 10% FBS, at 37 °C in humidified 5% CO_2_. 24 h post-transfection, cells were washed in PBS and incubated with low serum (1%) for 5 h before treatment with 1 µM TM for 24 h. Cell lines were received from the group of Prof. Seufferlein (Ulm, Germany), and identity verified by STR-analysis.

### Transfection of siRNA

PA-TU-8988T or BXPC-3 cells were transfected using Invitrogen Lipofectamine RNAiMAX Transfection Reagent (Thermo Fisher Scientific), according to manufacturer’s instructions. Transfection of Accell non-targeting siRNA (Dharmacon) and self-designed siDDOST was performed at a final concentration of 50 nM. First pool of DDOST siRNA consisted of sequences CAACGUGGAGACCAUCAGUGtt and CAUCAACGUGGAGACCAUCtt. Second pool of DDOST siRNA consisted of sequences GACAAGCCUAUCACCCAGUAUtt, AUACAGUGUUCAGUUCAAGtt, CAUCAACGUGGAGACCAUCtt, GUAUGGUGUAUUCCAGUUUAAtt, GUGAUCCAGCAGCUCUCAAAUtt.

### RNA quantification

Total RNA of lysed cells was isolated and purified using the NucleoSpin RNA kit (Macherey–Nagel) according to the manufacturers protocol. Equal amounts of RNA were reverse transcribed into cDNA using the Omniscript RT Kit (Qiagen) according to the manufacturers protocol. The mRNA expression was detected using quantitative real-time polymerase chain reaction (qRT-PCR) with the Luna Universal SYBR Green Supermix (NEB) via an Realtime PCR system (Applied Biosystems) and the corresponding primers for *DDOST* forward and reverse primer sequences were 5′TTGGTACCCTTCGGCAGGAGGAGGAA 3′ and 5′AAAGGATCCTTTGAGGGCAACATCTCG 3′. The ribosomal protein *RPLP0* or human *B2M* was used as an endogenous control. All experiments were performed in triplicates and are displayed in ± SD.

### Western blot analysis

Cells were lysed in RIPA lysis buffer (50 mM Tris–HCl (pH 7.5), 150 mM NaCl, 0.1% SDS, 1% sodium deoxycholate and 1% Triton X-100), supplemented with 4% Complete Protease-Inhibitor Cocktail (Roche) and 1% phosphatase inhibitor Mix I (Serva). After brief sonification, 20 µg of cell lysates were analyzed by SDS-PAGE (10% gels) and transferred onto PVDF membranes, followed by treatment with the appropriate primary antibodies against DDOST (HPA046841, Atlas Antibodies), β-Actin (A1978, Sigma-Aldrich) and the suitable peroxidase-conjugated secondary antibody (GE Healthcare). Target proteins were visualized using WesternBright Chemiluminescence Substrate Sirius system (Biozym). For reprobing, blots were stripped with Restore Western Blot Stripping Buffer (Thermo Scientific) before the addition of a new primary antibody. β-Actin antibody was used as loading control. Gels were scanned using INTAS Advanced fluorescence Imager and software ChemoStar version 0.4.21. Uncropped western blots, some cut prior to hybridization, are shown in Fig S2.

### Mass spectrometry

#### Sample preparation and TMT labeling

Five biological replicates of 100 µl cell lysates (1 μg/μl) total protein were collected and further processed for liquid chromatography-tandem mass spectrometry (LC–MS/MS) analysis following an adapted filter-aided sample preparation (FASP) protocol^45^. The protein samples were reduced with 10 mM TCEP (8 M urea in 50 mM HEPES, pH 8.5) and subjected to centrifugation with 30-kDa cutoff filtration units (Amicon). For alkylation, samples were incubated in the dark with 50 mM iodoacetamide in 8 M urea, 50 mM HEPES, pH 8.5, centrifuged and washed before trypsinization with 2 µg trypsin (Promega) in 50 mM HEPES, pH 8.5 at 37 °C overnight. For quantitation, tryptic peptides were labeled using the amine-reactive TMT10plex Isobaric Label Reagent Set (Thermo Fisher Scientific) following the manufacturer’s instructions.

#### Phosphopeptide enrichment

For phosphopeptide enrichment analysis, 980 µl of labeled peptide samples (see above) were purified using a high-select TiO_2_ phosphopeptide enrichment kit (Thermo Fisher Scientific) according to manufacturer’s protocol.

#### LC–MS/MS analysis

Peptide solutions were analyzed by LC–MS/MS via the Ultimate 3000 RSLC nano-HPLC system coupled to an Orbitrap Fusion mass spectrometer with an EASY-Spray ion source (Thermo Fisher Scientific). Samples were loaded on reversed-phase (RP) C18 pre-column (Acclaim PepMap, 300 μm × 5 mm, 5 μm, 100 Å, Thermo Fisher Scientific), samples were washed with 0.1% TFA before the peptides were separated on a 50-cm µPAC C18 separation column (PharmaFluidics). Peptides were eluted with a linear 360-min gradient ranging from 3 to 35% (v/v) acetonitrile containing 0.1% formic acid at a flow rate of 300 nL/min. For data-dependent acquisition (DDA), MS/MS experiments were performed for analyzing enriched phosphorylated peptides, while MS^3^ experiments were used for TMT-labeled peptides. For MS/MS, high energy collision-induced dissociation (HCD) was applied using normalized collision energies (NCE) of 27, 28 and 38%, as well as collision-induced dissociation (CID) using 35% NCE. The high-resolution full MS scans were followed by high-resolution product ion scans in the orbitrap and low-resolution scans in the linear ion trap. For MS^3^ experiments, CID was applied at 35% NCE. The high-resolution product ion scans were acquired in the orbitrap after simultaneous selection and fragmentation (HCD at 55% NCE) of the 10 most intense MS/MS fragment ions. For both modes, MS/MS and MS^3^, dynamic exclusion was enabled. Data acquisition was performed via the Xcalibur version 4.3 software (Thermo Fisher Scientific).

#### MS data analysis

For peptide identification and quantification, LC–MS/MS data were searched against the Swissprot database (taxonomy *Homo sapiens*, 04/23, 20,332 entries) using the SequestHT database search algorithm with Proteome Discoverer (version 2.4; Thermo Fisher Scientific). A maximum mass deviation of 10 ppm was applied for precursor ions while for product ions, max. 0.6 Da (linear ion trap data) and 0.02 Da (orbitrap data) were allowed. Oxidation of Met, acetylation of protein N-termini, and phosphorylation of Ser, Thr, and Tyr were set as variable modifications. Carbamidomethylation of cysteines and modifications of peptide N-termini and Lys by the TMT label were included as fixed modifications. A maximum of two missed cleavage sites were considered for peptides. Quantification was performed using the TMT reporter ion abundances derived from HCD spectra, reporter ion intensities of protein unique and razor peptides were added to give protein abundances.

For both, protein and peptide level analysis, instances with missing quantification for all replicates in both conditions were filtered out. For imputation of missing data, the K-nearest neighbors (kNN) algorithm using the ‘impute.knn’ function from the ‘impute’ R package was applied in DDOST KD and control KD experiments separately^46,47^.

Non-unique phosphopeptides and peptides with ambiguous phosphorylation sites were filtered out before phosphopeptide quantitation was performed. To correct for abundance differences at the protein level, phosphopeptides were normalized after log2 transformation by subtracting the corresponding log2 protein abundance for each replicate/condition, where given. To simplify data analysis, peptides indicating equal phosphorylation sites were merged by summing their reporter ion intensities.

### Proliferation assay

Cell proliferation was determined by cell counting using the CASY counter system (Omni Life Science). For KD-experiments cells were seeded in 24-well plates at a density of 25,000 cells / well in triplicates for each time point. Cells were harvested 24 h, 48 h and 72 h after seeding and absolute cell count was determined.

### ATP concentration assay

ATP concentration was measured with the CellTiter-Glo Luminescent Cell Viability Assay (Promega) according to the manufacturer’s instructions. Cells were seeded in 96-well plates at a density of 5,000 cells / well with five replicates and incubated 48 h post transfection at 37 °C. Finally, the luminescence of each well was measured by Luminoskan Ascent (Thermo Scientific).

### Immune fluorescence ER-stress

PDAC cells were grown on polylysine-coated coverslips in 24 well plates at a density of 50,000 cells / well and incubated 24 h post transfection at 37 °C in humidified 5% CO_2_. As positive control for ER-stress, 1 µM of TM was added. After incubation, cells were fixed with 4% paraformaldehyde for 15 min and permeabilized with 0.3% Triton X-100 (Sigma-Aldrich) in PBS for 10 min. Subsequently, cells were blocked with 5% goat serum (Thermo Fisher Scientific) in PBS for 1 h. Afterwards, cells were incubated with the primary antibodies for DDOST (HPA046841, Atlas Antibodies) and CHOP (2895, Cell Signaling) at 4 °C overnight, followed by incubation with Alexa Flour 488 and 594 labelled secondary antibodies (Thermo Fisher Scientific). Then, cells were mounted using ProLong Gold with DAPI (Thermo Fisher Scientific) and visualized using a wide-field fluorescence microscope (BZ-X810, Keyence). The area of CHOP-positive cells was quantified using ImageJ software. Three randomly selected images from each culture condition were analyzed.

### Intracellular ROS assay by flow cytometry

PDAC cells were grown in 12 well plates at a density of 100,000 cells / well and incubated 24 h post transfection at 37 °C in humidified 5% CO_2_. Intracellular reactive oxygen species (ROS) were detected using H_2_DCF-DA (HY-D0940, MedChemExpress). Briefly, cells were incubated with H_2_DCF-DA for 2 h at 37 °C in humidified 5% CO_2_. After washing with PBS, cells were incubated with propidium iodide (PI) for another 15 min and subsequently analyzed via flow cytometry (LSRFortessa, BD Bioscience; FlowJo Software (v7.6.5), BD Bioscience).

### Apoptosis assay by flow cytometry

PDAC cells were grown in 12 well plates at a density of 100,000 cells/well and incubated 48 h post transfection at 37 °C in humidified 5% CO_2_. After harvest, cells where resuspended in binding buffer and incubated with BD AnnexinV-FITC (556420, fisher scientific) and PI solution (P3566, Invitrogen) according to the manufacturer’s instructions. Subsequently, the cells were analyzed via flow cytometry (LSRFortessa, BD Bioscience; FlowJo Software (v7.6.5), BD Bioscience).

### Statistical analysis

LC–MS/MS protein- and peptide-level data were analyzed using the reproducibility-optimized test statistic (ROTS) test implemented in the R package ROTS, a non-parametric test^48^. Before performing the ROTS test, 1 was added to all abundances and the data were log2-transformed. Data gathered by western blot method, PCR and phenotypical assays are expressed as the mean ± SD. Statistical analysis was performed using Graph-Pad Prism (version 9.4.1). Differences were calculated using unpaired, two-tailed student’s t-test and considered statistically significant when *P* ≤ 0.05. *p*-Values of * *P* ≤ 0.05, ** *P* ≤ 0.01, or *** *P* ≤ 0.001 are indicated in figures. Spearman correlation analysis was performed on 03/27/2023, using GEPIA 2 analysis tool, based on the TCGA Tumor dataset of PAAD (pancreatic adenocarcinoma) cancer type^49^. *ρ*-results were normalized to *RPLP0* and interpreted according to Cohen (1988). Strong correlation: *ρ* ≥ 0.5, medium correlation: *ρ* ≥ 0.3, weak correlation: *ρ* ≥ 0.1 (n = 179).

### PPI and GADO network construction

Protein–protein interaction (PPI) clusters were created using the STRING database version 11.5 on 03/25/2023 (https://string-db.org/cgi/input.pl). Thereby, known and predicted protein–protein association data are collected and integrated. Both physical and indirect, functional interactions are associated as long as they are specific and biologically meaningful^50^. The STRING database was used to create the PPI network of DDOST with a minimum interaction score of 0.15 in the organism of homo sapiens including the whole genome as statistical background. The interaction predictions were obtained from databases, experiments, gene neighborhood, text mining and co-expression. The enrichment function of the GADO tool^23^ (https://www.genenetwork.nl/) which leverages gene co-regulation to improve prediction of pathway membership, was used to identify significantly enriched pathways in the set of 26 identified DEPs (Database: GO_P, Test type: wilcoxon, gene set analysis results downloaded: 2024-05-13).

### Supplementary Information

Supplementary Figures.

Supplementary Tables.

## Data Availability

Proteomics data generated in this work are available via PRIDE (PRoteomics IDEntification Database, https://www.ebi.ac.uk/pride/) using the identifier PXD047441.
