# Supplementary material for: Induction of oxidative- and endoplasmic-reticulum-stress dependent apoptosis in pancreatic cancer cell lines by DDOST knockdown
Source: Sci Rep. 2024 Sep 2;14:20388. doi: 10.1038/s41598-024-68510-8 (PMC11369111; doi:10.1038/s41598-024-68510-8)
Supplement: Supplementary file 1 — Supplementary Figures. [file 41598_2024_68510_MOESM1_ESM.pdf]

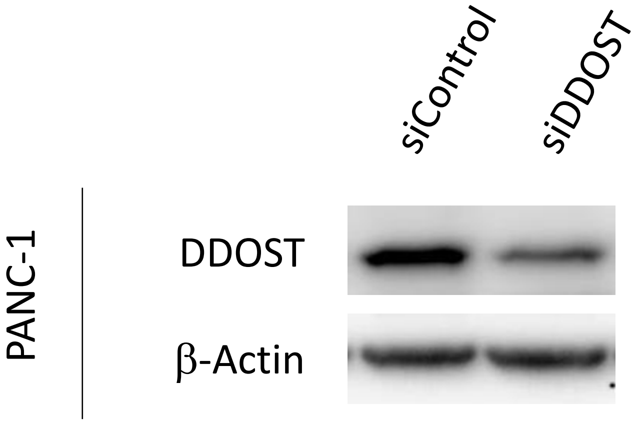

Figure S1. Western blot analysis of DDOST expression in PANC-1 cell line. β-Actin was used as loading control.

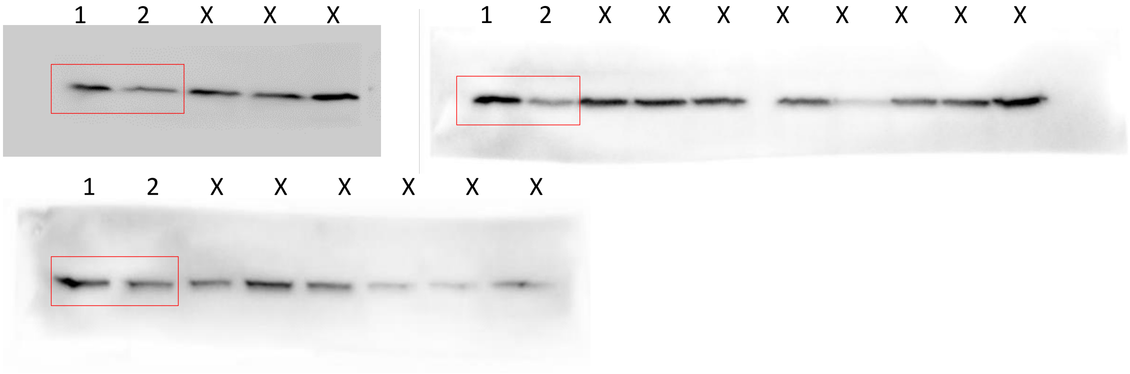

Related to Fig 2 A. BXPC-3 cell protein extracts incubated with DDOST antibody. Respective red boxes (lanes 1-2): repeated knockdown experiments shown in **Fig 2 A.** Lanes X: Not included in manuscript.

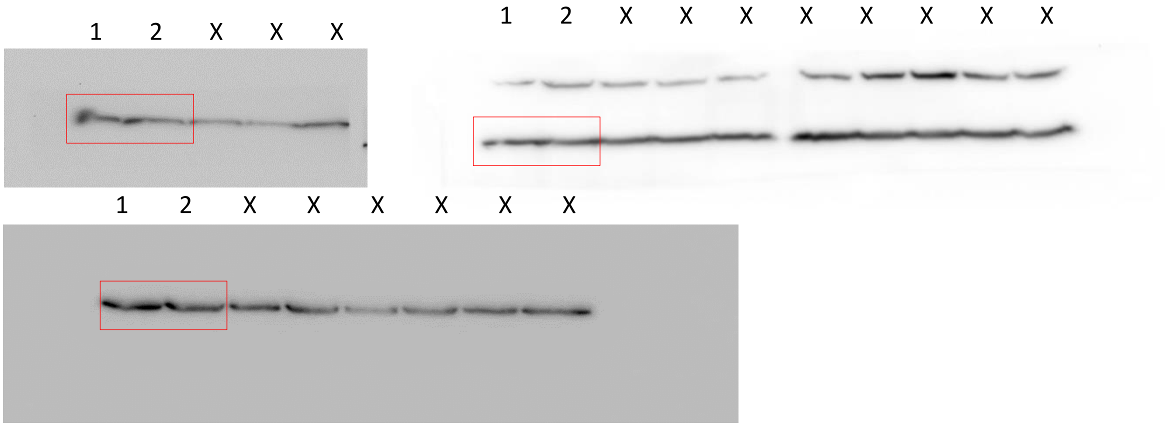

Related to Fig 2 A. BXPC-3 cell protein extracts incubated with β-Actin antibody. Respective red boxes (lanes 1-2): repeated knockdown experiments shown in **Fig 2 A.** Lanes X: Not included in manuscript.

Figure S2 (page 1 of 5). Raw images for the respectively indicated related Figures. Protein extraction and Western Blot was performed as described in material and methods section.

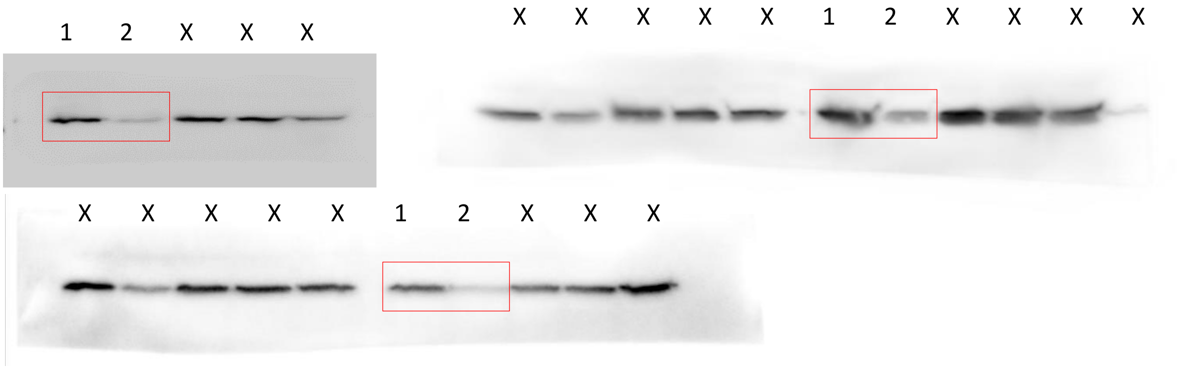

Related to Fig 2 A. PA-TU-8988T cell protein extracts incubated with DDOST antibody. Respective red boxes (lanes 1-2): repeated knockdown experiments shown in **Fig 2 A.** Lanes X: Not included in manuscript.

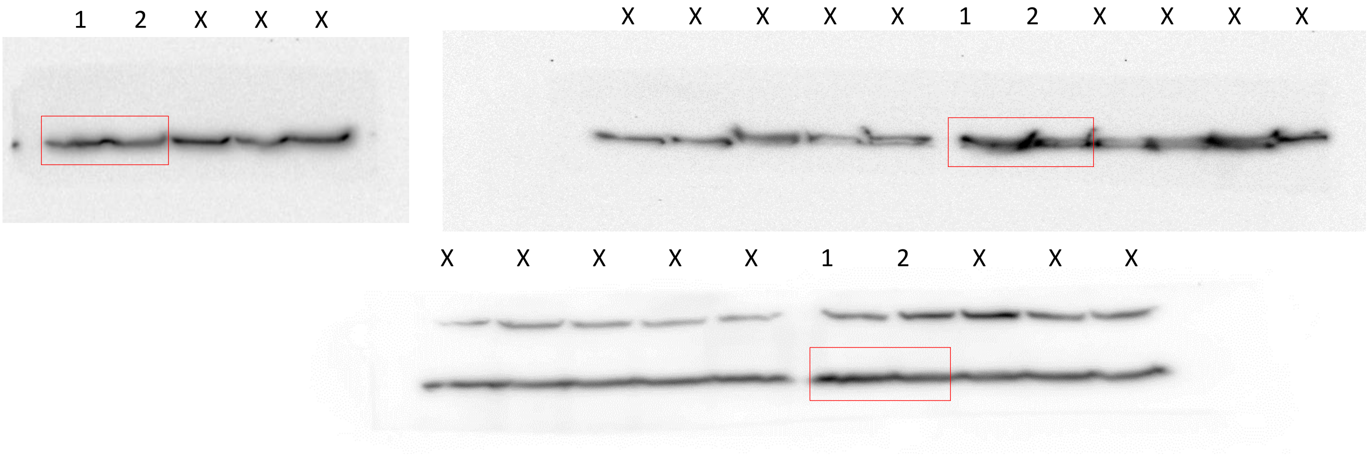

Related to Fig 2 A. PA-TU-8988T cell protein extracts incubated with β-Actin antibody. Respective red boxes (lanes 1-2): repeated knockdown experiments shown in **Fig 2 A.** Lanes X: Not included in manuscript.

Figure S2 (page 2 of 5). Raw images for the respectively indicated related Figures. Protein extraction and Western Blot was performed as described in material and methods section.

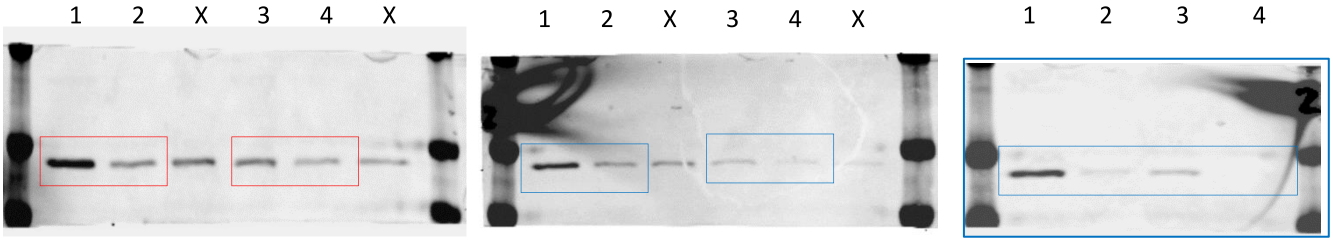

Related to Fig 4 G. BXPC-3 cell protein extracts incubated with DDOST antibody. Red boxes (lanes 1-4): experiment shown in **Fig 4 G.** Blue boxes (lanes 1-4): repeated knockdown experiments. Lanes X: Not included in manuscript.

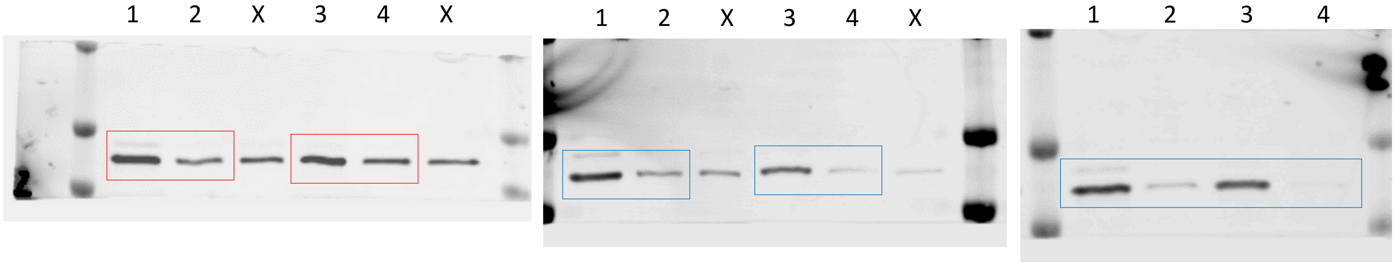

Related to Fig 4 G. BXPC-3 cell protein extracts incubated with β-Actin antibody. Red boxes (lanes 1-4): experiment shown in **Fig 4 G.** Blue boxes (lanes 1-4): repeated knockdown experiments. Lanes X: Not included in manuscript.

Figure S2 (page 3 of 5). Raw images for the respectively indicated related Figures. Protein extraction and Western Blot was performed as described in material and methods section.

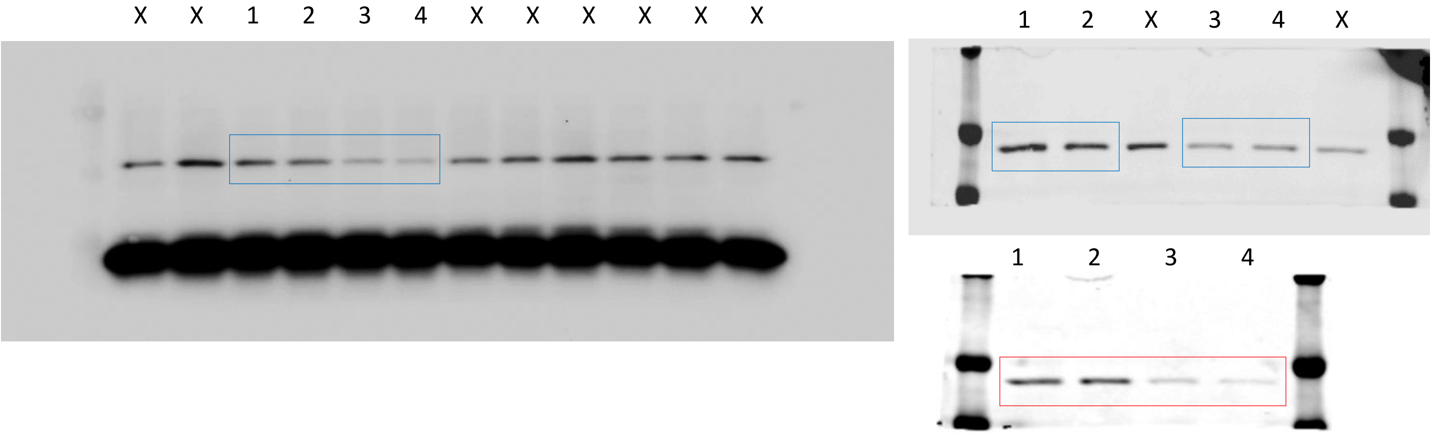

Related to Fig 4 G. PA-TU-8988T cell protein extracts incubated with DDOST antibody. Red boxes (lanes 1-4): experiment shown in **Fig 4 G.** Blue boxes (lanes 1-4): repeated knockdown experiments. Lanes X: Not included in manuscript.

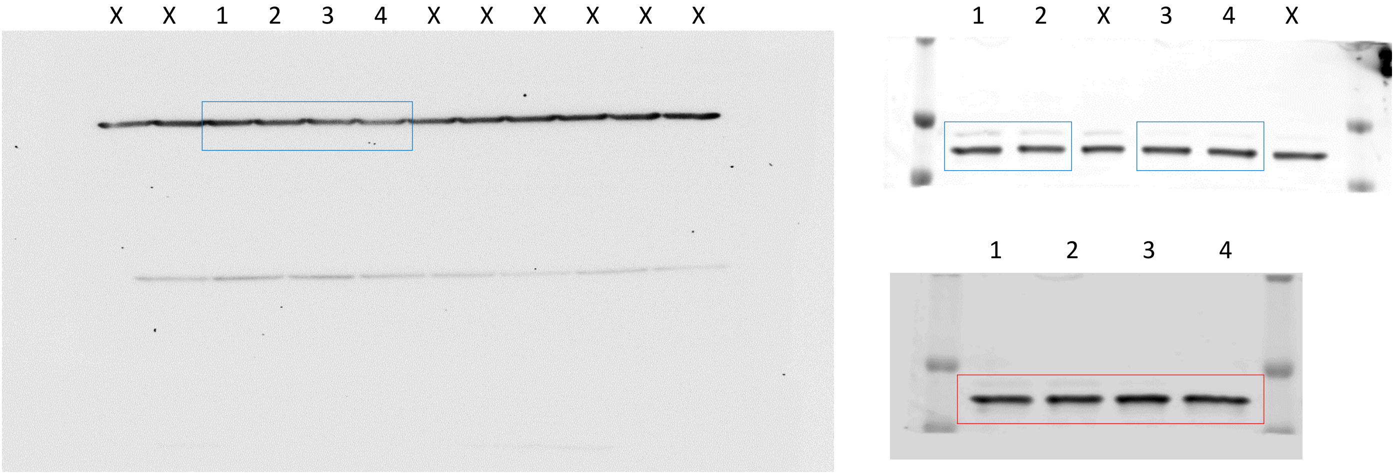

Related to Fig 4 G. PA-TU-8988T cell protein extracts incubated with β-Actin antibody. Red boxes (lanes 1-4): experiment shown in **Fig 4 G.** Blue boxes (lanes 1-4): repeated knockdown experiments. Lanes X: Not included in manuscript.

Figure S2 (page 4 of 5). Raw images for the respectively indicated related Figures. Protein extraction and Western Blot was performed as described in material and methods section.

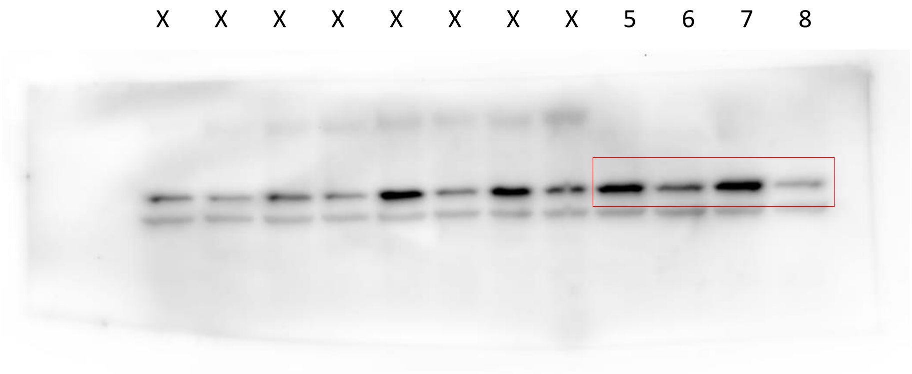

Related to Fig S1. PANC-1 cell protein extracts incubated with DDOST antibody. Red box (lanes 5-8): experiment shown in **Fig S1.** Lanes X: Not included in manuscript.

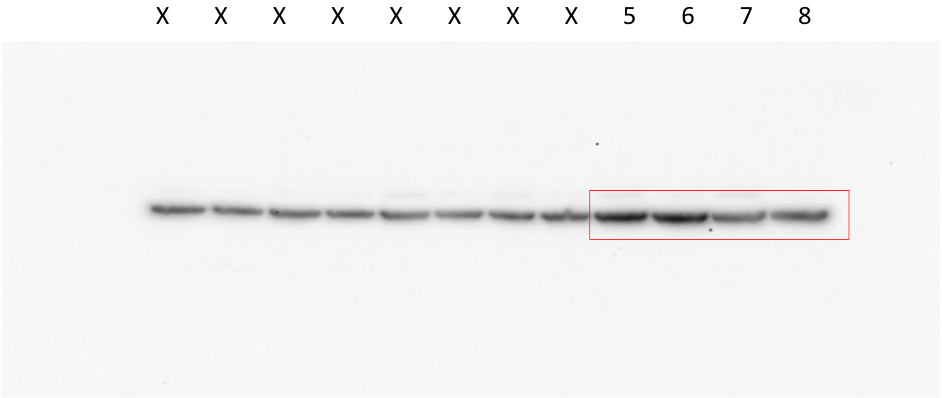

Related to Fig S1. PANC-1 cell protein extracts incubated with β-Actin antibody. Red box (lanes 5-8): experiment shown in **Fig S1.** Lanes X: Not included in manuscript.

Figure S2 (page 5 of 5). Raw images for the respectively indicated related Figures. Protein extraction and Western Blot was performed as described in material and methods section.
